# Peptides blocking ApoE pathological function safely reduce pathology in TgSwDI/apoE2, 3 and 4 AD mice

**DOI:** 10.1002/alz70859_102051

**Published:** 2025-12-25

**Authors:** Fernando Goni, Ding Wang, Jianina Suazo, Jasmine Lin, Jasmine Okeagu, Begonia Gamallo‐Lana, Adam Mar, Thomas Wisniewski

**Affiliations:** ^1^ NYU Grossman School of Medicine, New York, NY USA; ^2^ New York University School of Medicine, New York, NY USA; ^3^ New York University Grossman School of Medicine, New York, NY USA

## Abstract

**Background:**

We previously demonstrated that a peptoid (LPFFA) prevents Aβ binding to human apoE producing cognitive rescue and reduced Aβ pathology on AD mouse models. We have now developed specific peptides that disrupt α‐helices essential for apoE conformation and pathological function; and tested, in cerebral amyloid angiopathy (CAA) TgSwDI models knockin (KI) for human apoE2, 3 or 4; the efficacy and safety of the peptoid in combination with two peptides (127 and 148) that bind, without overlapping, the 120‐170 region of all human apoE isoforms.

**Method:**

For each TgSwDI, TgSwDI/apoE2, TgSwDI/apoE3 and TgSwDI/apoE4 model; two groups of 10 months animals were used for the preclinical trial. Each group was inoculated IP weekly for three months with the combination peptoid+ peptides 127/148 at 100 µg/animal/compound in sterile saline or sterile saline vehicle alone. Then, animals underwent locomotor and cognitive testing followed by brains being harvested for immunohistochemical and biochemical analysis.

**Result:**

The peptoid/peptides treated groups showed, irrespectively of the apoE background, significant cognitive rescue compared to controls on Barnes Maze; a significant decrease of vascular and parenchymal Aβ pathology, with similar or reduced microhemorrhages and activated glia shown by Iba1, GFAP and CD‐45 immunohistochemistry. Greatest behavioral improvement was shown in Tg mice with apoE4 KI, although the end point was similar to that of apoE3 KI animals.

**Conclusion:**

We have shown that diminishing Aβ binding to apoE and disrupting the pathological conformation of any apoE allele; significantly reduces Aβ pathology without secondary effects; constituting a safe and novel methodology to prevent early deposition and progression of Aβ pathology in AD Tg models, without associated ARIA like complications.

